# Methodological implications of adapting and applying a web-based questionnaire on health problems to adolescent football players

**DOI:** 10.1186/s12874-021-01406-7

**Published:** 2021-11-15

**Authors:** Solveig E. Hausken-Sutter, Astrid Schubring, Stefan Grau, Klara Boije af Gennäs, Natalie Barker-Ruchti

**Affiliations:** 1 Department of Food and Nutrition, and Sport Science, University of Gothenburg, Box 300, SE405 30 Gothenburg, Sweden; 2 Department of Sports Sciences, Malmö University, Malmö, Sweden; 3 School of Health Sciences, Örebro University, Örebro, Sweden

**Keywords:** OSTRC-H, Youth, Survey, Methodology, Prospective study

## Abstract

**Background:**

The Oslo Sport Trauma Research Centre Questionnaire on Health Problems (OSTRC-H) has become a popular tool to monitor health status in athletes. Originally developed for adult athletes, the tool is today also being used in adolescent athletes. However, little is known on the suitability of the questionnaire for the adolescent age group and the methodological implications of applying the tool to prospectively monitor illness and injury. To address this gap in methodological knowledge, the aim of this study is to outline and discuss the adaption and application process of the OSTRC-H to adolescent football players.

**Method:**

The adaption process included a slightly modified back-translation method to translate the questionnaire. The application process included a web-based version of the Swedish OSTRC-H sent out once a week over 23 weeks to 115 adolescent football players aged 10-19 attending two football schools in Sweden. The response rate and prevalence of health problems over 23 weeks were calculated as feasibility indicators. Additionally, comprehensibility questions were added to the questionnaire in the end of the study.

**Result:**

No major disagreement was found between the original and translated versions of the questionnaire. However, significant changes to the wording of the questions and answer categories were necessary to adapt it to adolescents. A visual body figure was also added. The average weekly response rate was 38% (SD 13.5). To increase this rate, questionnaire data was gathered retrospectively through telephone and email contact with the participants and their parents, elevating the response rate to 53% (SD 15.5). The adolescents experienced the questionnaire as easy to understand and to cover all relevant health problems.

**Conclusion:**

Our study demonstrates the importance of adapting the questionnaire to the adolescent target group through translation, pre-tests, adjustments of wording and the facilitation of answering the questionnaire using a visual body figure. The study further shows the importance of keeping close and personal contact with the participants, their parents, teachers, and coaches throughout data collection. Future studies should take into account the age group and study context when adapting and applying the OSTRC-H to adolescents.

## Introduction

To better understand and prevent injury development among adolescent athletes, monitoring their health status has in recent years gained importance [1–3]. For health monitoring to be successful, both the International Olympic Committee (IOC) and researchers have called for a more unified and evidence-informed approach toward the health of young athletes [4–7]. Two increasingly popular monitoring tools are the Oslo Sports Trauma Research Center Questionnaire on Health Problems (OSTRC-H) and the Oslo Sports Trauma Research Center Overuse Injury Questionnaire (OSTRC-O). The questionnaires have been developed to identify and record different types of health complaints including illness and injuries, and to quantify the effect these have on sport participation [8, 9]. Several studies have explored the psychometric properties of the questionnaires across different contexts and populations [7, 10–14], confirming that the questionnaires are valid and reliable tools. Additionally, a range of elite sport organizations have employed the tools in their sport injury research and clinical health monitoring programmes [15]. In recent years, the two questionnaires have also been applied to children and adolescents of different sport contexts [1, 2, 6, 7, 16–18]. Most of these studies, however, applied the questionnaires without reporting any consideration regarding methodological suitability and implications for application. This is problematic because throughout childhood and adolescence, language, literacy, and memory are under constant development and may therefore affect young research participants’ ability to answer survey questions [19]. Pretesting questions for their suitability with specific age groups is thus highly adivsable as this will affect data quality [19, 20]. With regard to the OSTRC-H questionnaire, Clarsen et al. [15] have in their latest revision of the tool emphasized the importance of adjusting the questionnaire when applying it to adolescents. Some of the studies that have applied the OSTRC-O/H to younger athletes have reported adaptions, such as language adaption [7] and supplemental interviews [2]. However, these studies did not report on the application process, hence did not focus on the study context (i.e. conducting the study through a sport club or schools) and specific administration procedures (i.e. text messages, email, app) when applying questionnaires originally developed for adult (elite) athletes. To further advance our knowledge on how choices of research design and methodology impact research participation and study outcomes, the aim of our study is to outline and discuss the adaption and application process of the OSTRC-H to adolescent football players.

## Material and methods

This study is part of the larger interdisciplinary research project “Injury-free children and adolescents: Towards best practice in Swedish sport” (FIT project), which is detailed in Hausken et al. [21] The project is an ongoing project that started in January 2017 and received ethical approval from the regional vetting board. The FIT project aims to produce a comprehensive picture of injury aetiology in a sample of male and female Swedish football players aged 10–19 years through integrating natural and social science and producing quantitative and qualitative data. In the present paper, we focus on the OSTRC-H questionnaire study, which was conducted from January to June 2018.

### The OSTRC-H questionnaire

**Fig. 1:**
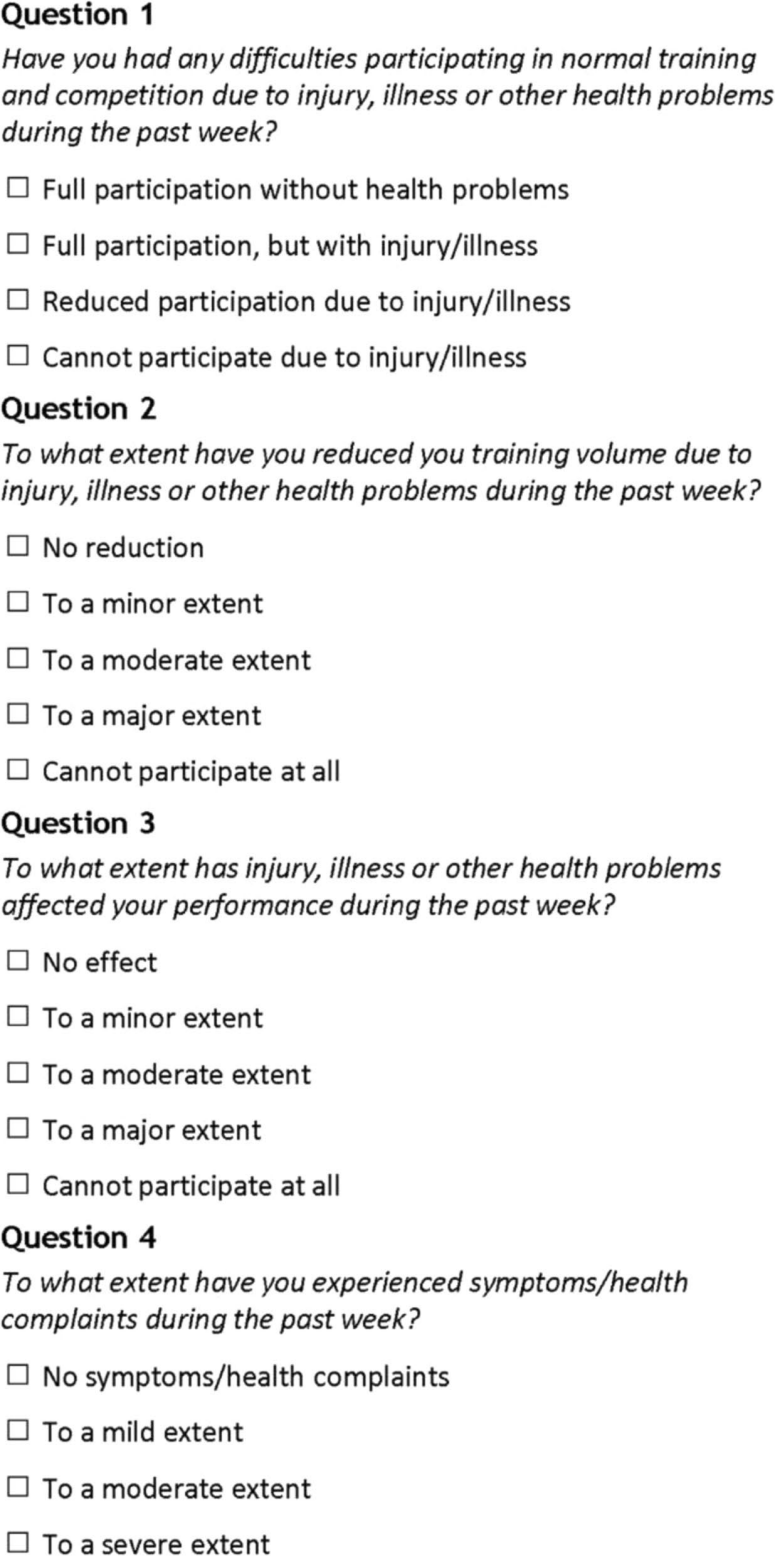
The four key questions asked at the beginning of the original weekly online OSTRC-H questionnaire

The OSTRC-H consists in its original form of four key questions that capture all types of health problems, including illness and injuries, their effect on sports activity (volume and performance) and intensity of symptoms (Fig. 1) [9]. Subsequent questions allow the respondents to report whether the problem they have referred to is an illness or an injury. In the case of an injury, respondents can register the area of the body in which it is located, and in the case of an illness, they can select major symptoms. For all types of problems, respondents are also asked to register (a) the number of days of complete training time loss; (b) reception of medical attention; (c) previous reporting of the problem; and (d) if so, place of reporting [9].

### Adaption process of the OSTRC-H

Approval to translate the OSTRC-H into Swedish and adapt it to adolescent football players was obtained from the developers. The English published version of the OSTRC-H and the Norwegian version, which the developers had created parallel to the English version, were used for the Swedish translation procedure [9]. The translation procedure was inspired by Beaton et al.’s [22] guidelines as well as the principles of good practice outlined by the International Society for Pharmacoeconomics and Outcome Research [23]. The following four steps, which have also been adopted by previous OSTRC-O/H translation and validation studies, [10–14] were followed: (1) Forward translation; (2) back-translation; (3) expert committee review; and (4) pretest.

#### Forward translation

Two independent bilingual Swedish residents with Swedish as their birth language translated the English version into Swedish (T1 and T2). One translator was aware of the concepts being examined, the other not [22]. The Norwegian version of the questionnaire was translated into Swedish by one bilingual Swedish resident with Swedish as birth language (T3). The translated versions were compared and discussed in separate meetings with each of the translators to resolve any discrepancies and create a synthesized version (T-123) [22]. Unresolved queries were cleared with the original developers [9, 22].

#### Back translation

Two independent translators, who were experts in survey research and whose native language was Swedish, translated the Swedish version (T-123) back to English (BT1 and BT2). Neither of the translators were familiar with the purpose of the translation nor the OSTRC-H [22]. A Swedish resident with birth language Norwegian translated the agreed upon version (T123) into Norwegian (BT3).

#### Expert committee review

The English and Norwegian versions (BT1, BT2, BT3) were compared to the original versions and discussed by an expert committee consisting of the translators and the FIT project team members (one professor in biomechanics, two associate professors in sports science, one doctoral student in sport science and one research assistant in sport science). The expert committee discussed semantic equivalence (e.g. do the words mean the same thing between the languages?) and conceptual equivalence (e.g. do the words hold different conceptual meaning for adolescents compared to adults?). The originators were contacted by email with minor queries. Eventually, the expert committee agreed upon one single, pre-final version of the Swedish OSTRC-H questionnaire.

#### Pretest

We pretested the questionnaire on a target population to determine its face validity and to evaluate its online delivery [22]. Pretesting methods such as cognitive interviewing has, for instance, proven to be an effective stratetgy when developing questionnaires for children and adolescents [19, 20]. We recruited five adolescents aged 10-15 (three boys and two girls), all of whom participated in sports, to conduct cognitive interviews to examine if the pre-final version was understandable [23]. The interviewers were FIT project team members with expertise in qualitative research interviewing; the interviews were held in groups of two-three (one or two adolescents and one researcher). The interview/pretest included a paper version of the questionnaire to be able to take written notes after each question. To ensure that the Swedish version retained its equivalence in the adolescent context [22], the researchers asked the adolescents questions such as: “How do you interpret this word/question?” “Do you understand this word/question?” “Do you remember your training/competition and health problems during the last week?” The adolescents were also encouraged to talk freely (“think aloud”) about the questionnaire [19]. The interviews were 15-25 min long.

Following the pretest, the expert committee discussed the questionnaire and made additional changes. Any issues relating to the interpretation of the questionnaire, such as misunderstandings around specific terms and uncertainty around what to report, were documented in writing [23]. Due to the amount of changes, we decided to perform another pretest of the questionnaire 4 weeks later with three of the adolescents who performed the pretest. This second pretest also allowed us to pilot the electronic version of the questionnaire. Finally, we proofread and finalized the web-based version of the questionnaire.

### Application process of the OSTRC-H questionnaire

This study targeted all eligible football players between 10 and 19 years old attending two collaborating football schools in Gothenburg (*N* = 499). The recruitment and enrollment process is visualised in Hausken et al. [22], Figure 1, page 7. The two schools were a primary and upper secondary school for pupils aged 10 to 15 (grades 4-9) and a high school for pupils aged 16 to 19 (grades 10-13). Out of the 499 invited players, 174 agreed to participate in the questionnaire study and signed written informed consent forms (parents signed for participants under 15 years). Players were included regardless of whether they had present or previous injuries or health problems. In total, 59 players dropped out during the study, which resulted in a final sample of 115 players. The remaining participants’ age ranged from 10 to 19 years (*M* = 14.7, SD = 2.8) with 37 female and 78 male players.

The questionnaire was set up through the University of Gothenburg’s SUNET Survey software. We distributed the web link weekly over 23 weeks from January to June 2018. The participants received the link each Monday morning to either their school, private or parents’ email address, depending on which address they had provided in the consent form. We had an agreement with the schools that the teachers would put aside time for the players every Monday morning to fill in the questionnaire in class and help them out if they had problems. During the first 2 weeks in January, two FIT project team members visited the schools to assist the teachers and participants in filling in the questionnaire. Regular visits to the schools during the study were also carried out by the two project team members. Participants who indicated health problems/injuries were encouraged to visit their school’s medical staff or collaborating football rehabilitation clinic, where the problems were diagnosed and noted in the participants’ files. Every other week, the research team received the recorded injury diagnosis information in encrypted form.

If participants did not respond to the weekly survey after 4 days, a reminder was sent out. If players still did not respond within a week, the participants or their parents and teachers were contacted through email, phone, or school visits to fill in the missing questionnaire data retrospectively, as well as to encourage the participants to continue to complete the questionnaire. A further effort to collect data retrospectively was made between April and May 2018 (weeks 14-22), when we called/emailed the parents/participants and visited the schools.

Additionally, the following questions were added to the questionnaire sent out at the 23rd week of registration to assess feasibility of the questionnaire: (1) Do you consider the questions to be clear and easy to answer? (2) Do the questions cover all areas where possible health problems and injuries can occur? (3) Do you think the questionnaire lacks important questions regarding possible health problems, pain and/or injuries? and; (4) Do you think the questions were offensive? The five answer categories ranged from 1 = completely agree to 5 = completely disagree and were dichotomized into agree/do not agree. After each question, the players were offered space to add comments.

### Data analysis

The response rate after 23 weeks and the prevalence of health problems over 23 weeks were calculated as feasibility indicators. The prevalence of health problems was calculated each week by dividing the number of players reporting a health problem with the number of questionnaire respondents [8, 9]. The prevalence of substantial health problems was also calculated with substantial problems defined as those leading to moderate or severe reductions in training volume, or moderate or severe reduction in sports performance, or complete inability to participate in sport (i.e. problems, where the participants selected options 3, 4 or 5 in either question 2 or 3) [9]. The prevalence calculations included retrospective data and was reported as average and 95% confidence intervals (95% CI). Data were compiled and analyzed using Microsoft Excel software.

## Results

### Adaption of the OSTRC-H

**Fig. 2:**
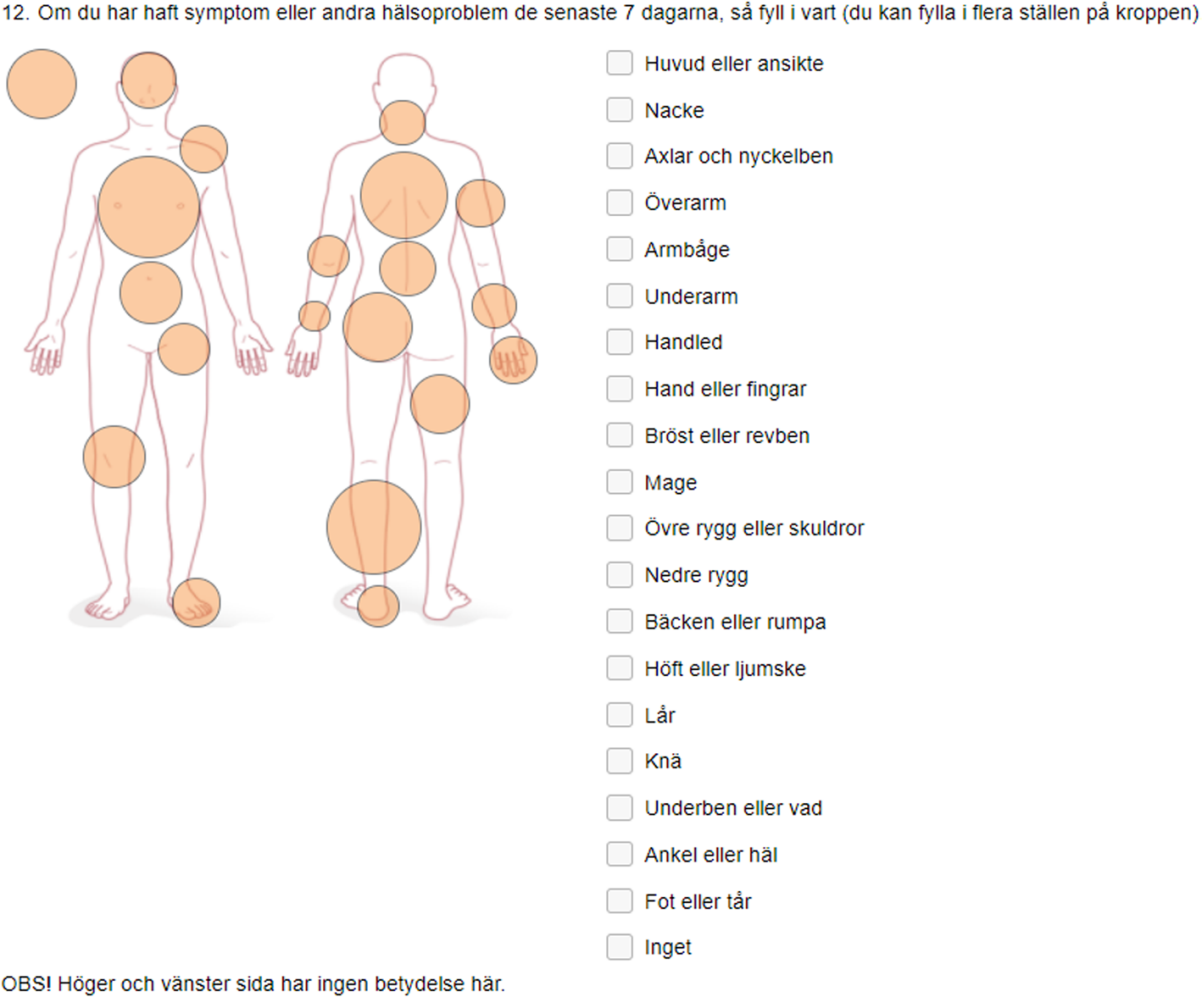
Body picture added to the Swedish version of OSTRC-H

When translating and adapting the OSTRC-H to the age group of adolescents we found no major disagreements during the translation process. After the expert committee review in step 3 of the translation and adaption process, the research team agreed to add questions to gather background data on sex, age, participation in other sports beside football, training hours per week (both football and other sports), number of training sessions and matches per week (football), and position on the field during training and match. Moreover, instead of the original question asking players to define whether the problem they referred to was an illness or an injury [12], we decided to ask: “Have you been sick during the last seven days (e.g. cold, cough or other)?” to better capture illness. If the participants answered yes to this question, they could freely note the illness they had experienced. We also added one more answer category to question 4 to enhance answer consistency: “Cannot participate at all”. Additionally, and as expressed by athletes in Ekman et al.’s [10] study, we decided to add a picture of a body shape to enable participants to indicate the specific body parts affected by physical problems (Fig. 2).

We found three major methodological implications after the pretests of the questionnaire. First, the adolescents had trouble understanding the term *past week*. The wording was therefore changed to “past seven days”, which has since also been changed in the OSTRC-H [15]. Second, the adolescents were unsure of what to report in terms of an injury (e.g. a sore finger or a broken leg). Uncertainty around reporting was solved by informing the participants in the beginning of the study that they should report all types of problems. Finally, the adolescents from the pretest had trouble remembering the training volume of the previous week. We therefore decided to follow the participants closely throughout the study.

**Fig. 3:**
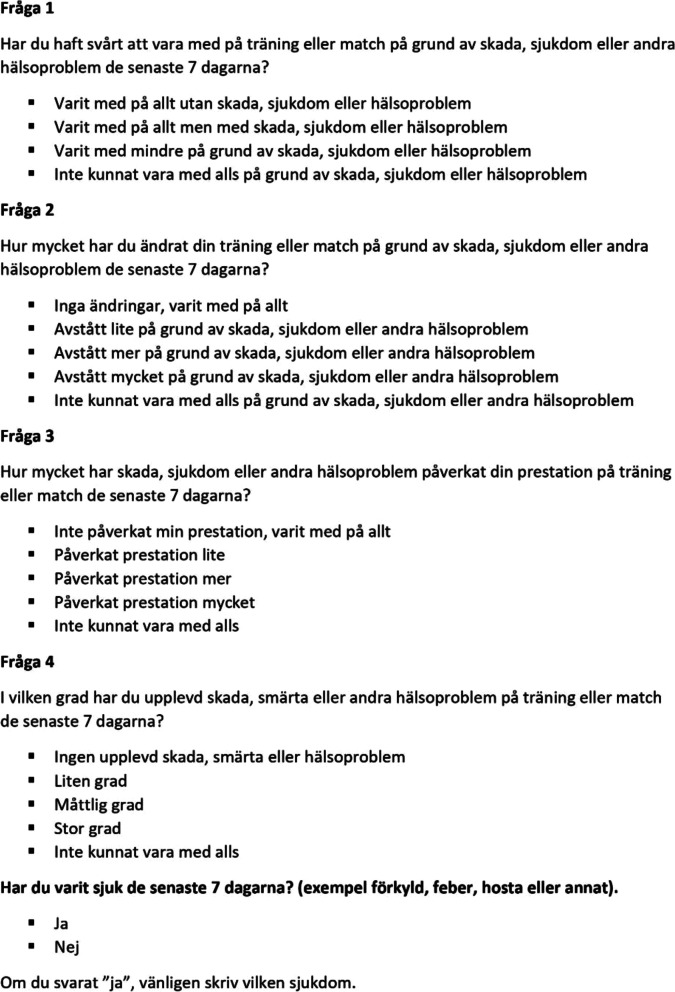
Our adapted and Swedish version of the four key questions asked at the beginning of the OSTRC-H questionnaire

Additionally, based on our questionnaire pretests, the following changes were made to the wording of the original four questions and their response categories to adapt the language to youth: *Question 1:* Removed the word “normal” and changed response categories 1 and 2 from “full participation” to “participated in everything”. We also aligned the response categories in question 1, i.e., we included “…injury, illness or other health problems” in every response category. *Question 2:* Changed the wording from “to what extent...” to “how much”. Additionally, instead of asking about how much participants had “reduced their training volume” we asked about the extent to which they had “changed their training or competition”*.* These changes are in line with the latest revision of the OSTRC-H [15]. Moreover, the phrase “…due to injury, illness or other health problems” was added to the response categories in question 2 after each response category to remind the adolescents that this was the most important aspect to report. Finally, the word “abstained” (in Swedish “avstått”) was added to answer category 2, 3 and 4 in question 2 as the pre-test adolescents understood this word better than “reduced”, which is the term used in the original questionnaire. *Question 3:* Changed the wording from “to what extent...” to “how much”. In addition, instead of asking about how much injury, illness or other health problems had affected their “performance”*,* we added “affected your performance in training or competition”. *Question 4:* Changed the word “symptom/health complaints” to “injury, pain or other health problems” due to the pretest participants struggling with the word “symptom”. We also added “training or competition” to show that we were interested in their problems in these contexts. Figure 3 shows our Swedish adapted version of the four key questions asked at the beginning of the weekly OSTRC-H.

### Application of the OSTRC-H

Table 1 shows the number of participants in each age group as well as the percentage of the total number of participants. The average response rate of each age group over 23 weeks is also presented.

**Table 1:** Overview of participants and response rates per school grade over 23 weeks, including retrospective data

**Grades**	**Number of participants**	**Percent of total sample (%)**	**Average response rate (%, SD)**
4 (10–11 years)	19	16.5	62 (21.4)
5 (11–12 years)	8	7	57 (20.6)
6 (12–13 years)	1	0.9	52 (51.1)
7 (13–14 years)	10	8.7	72 (13)
8 (14–15 years)	21	18	49 (14.3)
9 (15–16 years)	0	0	0
10 (16–17 years)	15	13	40 (15.4)
12 (17–18 years)	23	20	55 (14.5)
13 (18–19 years)	18	16	42 (18.6)
**Total**	**115**	**100**	**53 (15.5)**

**Fig. 4:**
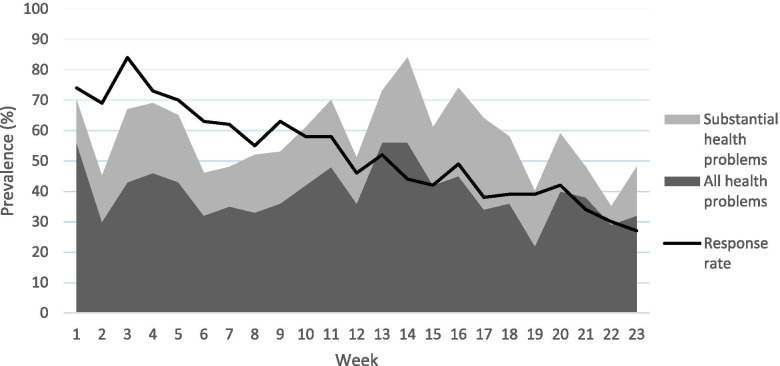
Response rate and weekly prevalence of health problems

The response rate of the overall sample over 23 weeks ranged from 25 to 74%, with an average response rate of 38%. The questionnaire data that we collected retrospectively through phone and email conversations over the 23-week-long period increased the average response rate to 53%, ranging from 27 to 83%. Figure 4 shows the response rate over 23 weeks (including retrospective data).

Figure 4 also shows the weekly prevalence of health problems and substantial health problems over 23 weeks for the adolescent football players. The average prevalence of health problems over 23 weeks was 40% [95% CI: 36-43%] with a prevalence of substantial health problems of 19% [95% CI: 16-21%].

Twenty-five players reported data on the last week of registration and were therefore eligible for the comprehensibility questions. All players agreed that the questionnaire questions were easy to understand and answer. One player commented that it was difficult to think back the last 7 days depending on which day during the week he/she completed the questionnaire. The player thought that “during the last week” would be better worded. All players agreed that the questions covered all areas of the body where possible injuries or health problems could occur. Seven players agreed that the questionnaire lacked important questions about possible health problems, pain, and/or injuries. One comment was that there should be an additional question on how the injury occurred (e.g., during football training or in another situation). None of the players experienced the questions as offensive.

## Discussion

The aim of this study was to outline and discuss the adaption and application process of the Oslo Sport Trauma Research Centre Questionnaire on Health Problems (OSTRC-H) to adolescent football players. To our knowledge, this is the first study to report on such issues when applying the OSTRC-H to adolescent athletes.

The results of the translation process showed that there were no major disagreements between the original English and Norwegian versions and the back-translated versions of the OSTRC-H. This result is in line with previous published translations of the OSTRC-H and OSTRC-O into Swedish [10], German [11], Danish [12], and Japanese [13, 14]. What we experienced as advantages of adapting and applying the OSTRC-H to adolescents is its simplicity, unambiguous and straightforward language, and its direct and specific questions [19]. What makes the tool challenging though is how the adolescents have to think back the last week remembering their training and health problems. We also found that considerable changes to the wording of the questions and answer categories were necessary when adapting the questionnaire to the study’s adolescent age groups. Finally, including a visualization of a body figure helped the participants to report health problems [10].

Although previous studies have demonstrated that the questionnaire is a valid and reliable tool [7, 8, 11–13], when adapting the questionnaire to contexts beyond adult sports, such as adolescent football, the wording needs to be adjusted and new psychometric testing and validation should be performed [15]. In alignment with other studies [10–12], we ensured feasibility through our pretest and the final questions about comprehensibility. The feedback we received through the pretest demonstrates the importance of testing the questionnaire in a target group through face-to-face conversational cognitive interviews. Further, it is important for the comprehensibility of the questionnaire to inform participating players in the beginning of the study about the questionnaire aims.

Regarding prevalence of health problems, we found that at any given time, 40% of the young football players experienced a health problem, and 19% experienced a substantial health problem. These numbers compare well with other studies on adolescent athletes [2, 17, 18, 24, 25], which have reported that approximately half of the reported health problems among adolescent athletes are substantial. In terms of response rate, we observed an overall low response rate. Comparable studies with the OSTRC-O or OSTRC-H have shown a higher response rate among adult athletes [10–14]. A higher response rate was also found in studies with younger athletes [1, 2, 6, 16–18, 24, 25]. Some of these studies, however, relied heavily on parental engagement [1, 18], weekly follow ups and methods to collect data retrospectively (e.g., interviews; weekly follow-ups) [2, 6, 18]. For other studies [10–12, 14, 24], the number of participants was significantly lower than in our study, which could have allowed the researchers to better accompany and follow up their participants. Furthermore, the application of the OSTRC-H/O differered throughout these studies. While some also distributed the questionnaire via email [2, 6, 11, 18], others used smartphone applications, SMS, phone interviews [1, 2, 7, 17], or other forms of message applications or platforms [12–14]. The larger sample size and the different application procedure could have affected the response rate of our study. Finally, in an updated version of the OSTRC-H, Clarsen et al., [15] suggested a new logic to reduce unnecessary responder burden by ensuring athletes only receive questions relevant to their current health state. Applying this logic could have encouraged the adolescents to answer the questionnaire.

Based on discussions in existing research and informal conversations with and feedback provided by our participants, their parents, teachers, school medical staff and coaches, several reasons help explain our study’s low and unstable response rate, and drop-outs. First, we experienced lower response rates during holidays (weeks 7, 13 and 14) and when players had long-term health problems. These findings are in line with Pluim et al. [24] who studied injuries and illness in elite junior tennis players. Second, we noticed that a large percentage of the 16-19-year-old players did not respond during some weeks, and many dropped out of the study during the first weeks. While we tried to counter non-responding and drop-outs by being present at school and keeping close contact with the participants, engagement of the oldest participants remained low as many reported feeling too busy with school and/or football or not finding responding meaningful. None of the oldest participants expressed any practical issues with the web-based registration method. The 10-13-year-old participants, in contrast, experienced several practical problems. Most importantly, they were unfamiliar with the school email system and either forgot to log in or had forgotten their address or password. Our agreement with the school’s teachers to put aside time for the participants once a week to fill in the questionnaire in class and help them if they had any problems was only successful during the first weeks, and not in all classes. Eventually, we engaged parents to support their adolescents in filling in the questionnaire. Parental support was also necessary in Pluim et al.’s [24] Leppänen et al.’s [1] and Schoeb et al.’s [18] study. It is, however, possible that parents may not have been aware of their adolescent’s health problems, which can have affected the results. Finally, we also see a relation between adolescent players’ demands in school and low participation and answer rates as it was the case for participants in grade 6 (12-13-year-olds), which were in their finale term of primary school and participants in grade 9 (15-16-year-olds), which were in their final term of secondary school in Sweden.

As Clarsen et al. [15] emphasized, a close and personal interaction with the participants is crucial to motivate them to complete the questionnaire weekly. We noticed, for example, that the response rate increased in the weeks during and/or after our visits to the schools (e.g., weeks 3, 9, 11, 16 and 20; see Fig. 4). In our study, we relied upon school managers, teachers and medical staff to support the application of the questionnaire, a choice reflective of research personnel resources. However, with the benefit of hindsight, we realize that we could have increased the response rate through controlling the weekly application of the questionnaire ourselves.

In terms of limitations of our research on the adaption and application process of the OSTRC-H to adolescent football players, our explanations for the drop-outs from the study and the low and unstable response rate rely on discussions in existing research and informal conversations with our participants and associated others. While the role of informal conversations in qualitative social and educational research methodologies is contested, scholars argue that they add “context” and “authenticity” to data that allows “transferability”, which is a generalizability based on qualitative research demonstrating the extent to which results are transferable to other settings [26]. We suggest that our explanations for the drop-outs and response rate allow transferability to other research with adolescents. Finally, we did not assess the reliability of our OSTRC-H questionnaire. However, we did not expect that the changes in wording we had made would compromise the reliability that existing studies have shown [7, 11–13].

## Conclusion

This study demonstrates the methodological challenges of applying questionnaires originally made for adult elite athletes to adolescent athletes, necessitating careful adjustment and pretesting, preferably with cognitive interviewing, to ensure feasibility. The participants’ age group and the contexts also require careful consideration, especially regarding the application of the questionnaire. We thus recommend that researchers aiming to study injuries in adolescent sport participants adopt a systematic procedure such as presented in this article to translate and/or adapt the OSTRC-H. Further, our experiences have demonstrated that specific considerations should be given to the application procedure, esp. regarding the involvement of mediators (e.g., school and teachers; sporting clubs and coaches; parents). Finally, researcher presence throughout the application process is crucial for the quality of the data, the response rate, and data validity.

## Data Availability

The datasets used and analysed during the current study are available from the corresponding author on reasonable request.
